# 28 days later: A prospective daily study on psychological well-being across the menstrual cycle and the effects of hormones and oral contraceptives

**DOI:** 10.1017/S003329172400357X

**Published:** 2025-02-07

**Authors:** Anne Marieke Doornweerd, Lotte Gerritsen

**Affiliations:** 1 Department of Experimental Psychology, Utrecht University, The Netherlands; 2 Department of Clinical Psychology, Utrecht University, The Netherlands

**Keywords:** diary, estradiol, mood, oral contraceptives, progesterone, sex hormones, testosterone

## Abstract

**Background:**

We aimed to study how hormonal status (oral contraceptive [OC] users vs naturally cycling [NC]) affects different dimensions and variability of psychological well-being, and how they relate to sex hormone levels (estradiol, progesterone, and testosterone).

**Methods:**

Twenty-two NC participants and 18 OC users reported daily affective and physical symptoms and collected daily salivary samples across 28 days. Groups were compared using psychological well-being averages (linear mixed models), day-to-day variability (Levene’s test), and network models. Within NC participants, cycle phase effects and time-varying associations between hormones and psychological well-being were assessed using both person-centered mean and change (subtracting mean from daily score) scores.

**Results:**

Lowered variability was found for OC users’ agitation, risk-taking, attractiveness, and energy levels. They showed lower overall ratings of happiness, attractiveness, risk-taking, and energy levels (range *R^2^_m_ =* .004: .019) but also reported more relaxation, sexual desire, and better sleep quality (range *R^2^_m_ =* .005; .01) compared to the NC group. The impact of sex hormones on psychological well-being varied significantly across cycle phases, with the largest effects for progesterone levels.

**Conclusions:**

Our results confirm that hormonal status is associated with a range of psychological well-being domains beyond mood and sexual desire, including energy levels, feelings of attractiveness, risk taking, and agitation. Lowered variability in OC users versus NC participants fit with ‘emotional blunting’ as a possible mechanism behind OC’s side effects. Our findings that show the menstrual cycle and sex hormones differentially influenced markers of psychological well-being emphasize the need to adequately account for the menstrual cycle.

## Introduction

The complex relationship between hormonal changes during the reproductive years and psychological well-being highlights an important but often overlooked aspect of female mental health. The menstrual cycle has a debilitating effect on a minority of the ovulating population (3–8%) in the form of premenstrual dysphoric disorder (PMDD) (American Psychiatric Association, 2013) or as premenstrual exacerbation (PME) of symptoms of pre-existing psychiatric disorders (Pinkerton et al., 2010). In the general population, those with a menstrual cycle experience symptoms during their cycles more sub-clinically, as evidenced by widespread reports of premenstrual syndrome (PMS) symptoms (Johnson, 1987; Steiner, 2000). Approximately 75% of those with a menstrual cycle experience symptoms such as irritability, mood swings, depression, fatigue, and food cravings during the premenstrual week, the week before menstruation begins (Johnson, 1987; Steiner, 2000). These affective, behavioral, or physical symptoms occur in response to normative changes in the hormonal milieu (Sundström-Poromaa, 2018). The menstrual cycle starts on the first day of menstruation with low estradiol and progesterone levels. Estradiol levels rise and peak prior to ovulation. The post-ovulatory drop in estradiol marks the start of the luteal phase, and both estradiol and progesterone levels peak in the mid-luteal phase. If the ovulated egg is not fertilized, the hormone levels drop, the uterine lining is not maintained, and the next menstruation follows (Abraham et al., 1972).

Although a large proportion of those with a menstrual cycle report a worsening of well-being during the luteal phase, to which extent this can be explained by hormonal fluctuations is still unclear. Of 47 studies of mood and the menstrual cycle reviewed by Romans et al. (2012), only 25 found an association between the premenstrual phase and negative mood. More recent studies add to these conflicting findings. A longitudinal study across two menstrual cycles found no significant fluctuations in mean negative affect (Hengartner et al., 2017). In contrast, Pierson et al. (2021) confirmed a premenstrual decrease in happiness and sexual activity consistent across cultures, based on 241 million observations from a menstrual cycle app. Regardless, the consensus is that high estradiol levels positively affect mood (Sundström-Poromaa, 2018), with progesterone as the hormone that provokes a decrease in well-being in the luteal phase (Sundström-Poromaa et al., 2020). Testosterone is another sex hormone that peaks during ovulation and has potential anxiolytic and antidepressant effects but is often understudied in relation to female-specific well-being (McHenry et al., 2014).

The effects of sex hormones on well-being are thought to occur through their actions on the brain. They may directly influence brain structures and function that underlie mood regulation and emotional processing but also modulate mood-related neurotransmitter systems (Comasco & Sundström-Poromaa, 2015; Montoya & Bos, 2017). Estradiol affects dopaminergic (Yoest et al., 2015) and serotonergic (Frokjaer et al., 2015) systems that are relevant for reward processing and mood. Estradiol can also influence social behaviors by enhancing oxytocin functioning (Bos et al., 2012). Progesterone’s anxiogenic effects likely results through its metabolite allopregnanolone, a known modulator of g-aminobutyric acid (GABA) function, the brain’s main inhibitory neurotransmitter ((Lambert et al., 2009). Moreover, progesterone is converted into cortisol in the presence of stress. Testosterone is particularly relevant for the striatal reward system (Hermans et al., 2010) and may also affect serotonergic (Fink et al., 1999) and GABA-ergic functioning (Bitran et al., 1993).

Hormone fluctuations during the cycle are stabilized by taking daily doses of synthetic forms of estradiol and progesterone (progestins) in the form of oral contraceptives (OCs). OC use has also been associated with deterioration in psychological well-being, with 4–10% of users reporting mental health side effects, including depressed mood, anxiety, irritability, mood swings, and decreased sexual desire (Gingnell et al., 2013; Poromaa & Segebladh, 2012; Sanders et al., 2001). Randomized placebo-controlled trials have confirmed decreased well-being in OC users, but the effects were small, and no clinically relevant differences in mood were found (Bengtsdotter et al., 2018; Lundin et al., 2017; Zethraeus et al., 2017).

National registry and epidemiological studies can offer information on the population level and over a longer period of time. They found OC use to be associated with an increased risk of depression, antidepressant use (Skovlund et al., 2016; Zettermark et al., 2018), and suicide (Skovlund et al., 2018). However, in light of other similarly statistically powered studies that show null (Duke et al., 2007; Lundin et al., 2022) or positive effects (Cheslack-Postava et al., 2015; Doornweerd et al., 2022; Keyes et al., 2013; Toffol et al., 2011, 2012), the clinically significant effects of OC use seem to be driven by a minority of users and are small in the general population (Lewis et al., 2019; Robakis et al., 2019; Schaffir et al., 2016). The effects of OCs on subclinical measures beyond anxiety and mood have been studied less. The three randomized controlled trials on OC use give some insight, as they suggested (minor) increases in anxiety, mood swings, and irritation in the OC arm (Graham et al., 1995; Lundin et al., 2017; Zethraeus et al., 2017).

Examining not only negative influences but also positive markers of psychological well-being throughout the cycle may help to dispel the persistent stereotype that having a menstrual cycle is inherently linked to being ‘emotional’- and negatively so. Consistent with this, a prospective study found an increase in positive and a decrease in negative dimensions of mood during the periovulatory phase (the time around ovulation) (Hromatko & Mikac, 2023). The periovulatory window is furthermore associated with behaviors reflecting heightened reward sensitivity and risk-taking, such as assertiveness (Blake et al., 2017), increased positive mood, and sexual desire (Hromatko & Mikac, 2023; Krüger et al., 2023; Ocampo Rebollar et al., 2017). In addition, the broad effects of hormones extend beyond mood symptoms to behavior and physiology. For example, while mood or sleep reports decreased only slightly in the mid-luteal phase, Alzueta et al. (2022) found a moderate worsening of self-reported physical symptoms in the mid-luteal phase.

The limited understanding of hormone-related well-being may be due to methodological challenges in studying menstrual cycle effects, including the operationalization and number of observations during the menstrual cycle (Schmalenberger et al., 2021). The inherent intra- and inter-variability that comes with studying psychoneuroendocrinology means that markers of well-being may be differentially affected depending on the cycle phase and requires study designs such as daily diary studies that take this into account (Pritschet et al., 2021). Studies that rely on retrospective reports or cross-sectional designs are subject to bias, which is circumvented by the use of prospective measurements. For example, two studies found participants to retrospectively report a negative change in mood but showed minimal changes in prospective self-report ratings (Graham et al., 1995; Oinonen & Mazmanian, 2001). The daily diary approach to studying the effects of hormones and cycles on psychological well-being also offers new avenues for studying the complexity and time-varying nature of the topic.

For OC use, the daily diary method provides a way to consider OC effects on both levels and variability in psychological well-being. The blunting effect of OC use on endogenous levels of estradiol and progesterone is thought to be paralleled by a blunting effect on emotions (Oinonen & Mazmanian, 2002). In turn, emotional blunting is a common symptom in people with depression (Christensen et al., 2022). On the other hand, a more stable emotional internal environment during active OC use could prove beneficial to people who are sensitive to hormone-related mood swings. Indeed, OC use was associated with an improved mood compared to the perimenstrual phase (Lundin et al., 2017) and is used as a treatment for PMS symptoms and PMDD (Lopez et al., 2012; Pearlstein et al., 2005; Yonkers & Foegh, 2004). This was also shown by other prospective studies. Stable OC use was characterized by less mood variation than, for example, non-use or change in use, and paralleled by lower mean weekly negative mood (Ott et al., 2008). Hamstra et al., (2017) found reduced mood variability specific to the mid-luteal phase, with OC users having lower ruminating thoughts and interpersonal sensitivity, but no differences were found in positive or negative affect. Possibly, the focus on affect level rather than affect variability might explain the discrepancy between the high rates of mood side effects and inconsistencies in studies focusing on affect differences (Jarva & Oinonen, 2007).

This study aimed to gain a better understanding of (1) how OC users differ from those with a natural menstrual cycle in terms of psychological well-being levels and variability over 28 days, (2) how psychological well-being fluctuates across different phases of the menstrual cycle in naturally cycling participants, and (3) the contributions of sex hormones to psychological well-being at various menstrual cycle phases. Using a daily diary study, we assessed the relationship between daily self-reported well-being and daily salivary sex hormone levels (estradiol, progesterone, and testosterone). OC effects were evaluated by examining differences in psychological well-being across (1) average levels over time, (2) overall variability, and (3) network models of psychological well-being dimensions. In addition, in NC participants, the relationship between hormone levels and psychological well-being was explored using person-centered hormone levels (average and change scores) to predict well-being within specific menstrual cycle phases. Network models were also used to investigate the underlying associations between individual aspects of psychological well-being and hormone levels.

## Methods

### Participants

Participants were recruited from a local university campus using flyers, social media, and directly approaching students. This study was part of a bigger study investigating the impact of sex hormones on psychological well-being in those with a natural menstrual cycle, OC users, and males. Only students with female as biological sex (identified by self-reported sex assigned at birth) of reproductive age (between 18 and 35 years old) were included in the current sample. The exclusion criteria for both the NC group and OC users were a BMI outside the range of 19–30, a gynecological or medical condition affecting hormone levels, self-reported pregnancy or breastfeeding, reported use of hormonal medications other than contraceptives, and a reported change in psychotropic medication use in the 6 months before study onset. NC participants were included if they had, on average, 1 menstruation per month (a cycle length of 21–35 days) and did not use HCs in the 6 months before study onset. For OC users, participants were required to use their OC for 3 months or longer before the study onset; there were no criteria for OC type or androgenicity. Eleven OC users reported a pill inactive phase during the study. At study onset, 25 NC participants and 18 OC users were included, of which 3 NC participants were excluded due to incomplete hormone data, which resulted in a final sample of 40 participants. The data on the male participants were not included in this paper.

### Procedure

To be invited to an enrollment visit, participants had to complete an extensive online screening to check for eligibility. During the visit, eligible participants were instructed on the procedures and requirements of the study, and if still interested, completed informed consent procedures. Following the enrolment visit, participants received their saliva sample collection kits and were instructed again on correct saliva sample collection and storage. Baseline questionnaires were completed the day before daily well-being and saliva measurements started. Data for the daily measures were collected for 28 days, based on the average length of a menstrual cycle. The day of study onset was the same for every participant, and the total study spanned the period between mid-April and mid-May 2023. Participants were paid €150 for completing the entire study. All procedures contributing to this work comply with the ethical standards specified by the faculty’s ethical review board that the research was approved by (20–178).

### Measures

The baseline questionnaire included questions on demographics, sexual orientation, romantic relationship status and satisfaction, menstrual cycle information, and history of and current oral contraceptive use. Participants were sent daily email prompts at 7 am with the link to the online questionnaire to collect daily self-report measures and included a reminder to take the saliva sample. The survey could be completed using any electronic device and had to be completed (together with the saliva sample) before 12 pm. All questionnaires were administered using Qualtrics (Qualtrics International Inc.).

For the daily self-report questions, participants were asked to rate how they felt at the current moment by putting the slider near the feeling that best described their state. The *affect domains* that were included were depressed/sad – happy, stress/anxious – relaxed, irritable/angry – calm, unattractive – attractive, tired – full of energy, and impulsive/risk taking – cautious. In addition, participants were asked about their *physical symptoms* by marking their appetite (little – a large), sexual desire (little – a lot), and their quality of sleep (badly – well). The answers were recorded on a scale from 1:100. Finally, participants could indicate whether they took part in any of the following activities: sexual activity in any form (including masturbation/solo sex, physical exercise (cardio or strength training where your heart rate went up for >20 mins), drug use (weed, hash, XTC, cocaine, mushrooms, LSD, ketamine, etc.), alcohol use (of any amount of type), illness so bad you need to stay in bed. People who menstruated could indicate whether they started their period that day, and for OC users, whether they were in the pill-inactive phase on the day of completing the survey.

### Salivary hormone sampling and analysis

Participants were instructed to use a passive drool technique to collect daily saliva samples of estradiol, progesterone, and testosterone using SaliCaps (IBL-International GmbH), collecting at least 0.5 mL of saliva. Salivary samples were instructed to be timed between 7 am and 12 pm. Participants were asked not to eat, drink, smoke, chew gum, or brush their teeth 30 minutes before experiment onset, to wait at least 10 minutes after rinsing their mouth before taking the sample and to take the sample at the same time of day. Saliva samples were initially stored in home freezers and collected every 7 days to be stored in the faculty’s freezer at −27 °C for later analysis by the Dresden LabService. Liquid chromatography–mass spectrometry (LC–MS) was used for progesterone and testosterone (pg/mL), with estradiol being analyzed using immunoassays (ELISA, IBL International, REF 30121045, Hamburg, Germany). The inter- and intra- assay coefficients of variation were for estradiol 11% and 13%, progesterone 7% and 7%, and testosterone 4% and 9%.

### Statistical analysis plan

#### Menstrual cycle phase coding

On the basis of menstrual cycle information, each testing day can be assigned to a cycle day. In the baseline questionnaire, participants provided the date of the first day of their previous menstruation and average cycle length. The date of the next period was extracted during the study from the daily self-report activities. This information was used to assign testing days (days 1:28) to cycle days (depending on the cycle length and timing of the experiment with regards to the participant’s cycle). For these cycle days, we used a combination of forward counting (counting forwards from menses onset, day of menses onset is day +1) and backward counting (counting backward from the next menses onset, day −1 is the day before the next menses) (Schmalenberger et al., 2021). From there, we applied a combination of the Schmalenberger et al. (2021) recommendations and a data-driven approach to code cycle days for each participant into 5 cycle phases: perimenstrual phase, mid-follicular phase, peri-ovulation, early luteal phase, and late luteal phase (see Supplementary Figure S1 for further explanation).

#### Comparison between the OC and NC groups

The statistical analyses were done using R software, version 4.1.2 (R Core Team). To compare the two groups of participants (OC versus NC) on baseline descriptives, we used ANOVA models for continuous variables and Chi-squared tests for ordinal variables. To test the hypothesis that OC users show less fluctuation over time on each psychological well-being aspect, we used Levene’s test for variation differences. To test for level psychological well-being differences between the two groups, we used linear mixed models with the lmer function of the lme4 package (Bates et al., 2015) for each domain separately. The group variable (OC versus NC) was included as a fixed factor, day as a random factor, and we used a random intercept for each participant. To compare an equal number of measurements for the NC and OC groups, measurements during pill pause days were included in the analyses and were ordered in the last week of the 28-day study period for the participants who included a pill pause phase during the study.

##### Estimation of network models:

To examine the underlying relations between each psychological well-being variable, and potential group differences, network models were estimated across all psychological well-being aspects for the OC and NC groups separately. We estimated network models for the total 28 days of the study duration for the NC group, where data were ordered based on calculated menstruation day. For the OC group, only the active pill intake days were included in the network analyses. A graphical LASSO regularization was used, in which the network is estimated by estimating a sparse inverse of the variance–covariance matrix. This was coupled with EBIC model selection to increase network specificity and reliability (Epskamp & Fried, 2018). To bootstrap network estimation, we used the R package bootnet to enable the assessment of the spread of the parameter and centrality estimates. Based on bootstrapped network estimations, we plotted the centrality statistics and estimated the strengths of nodes and edges (Epskamp et al., 2018).

#### Associations between hormones and psychological well-being throughout the menstrual cycle in naturally cycling participants

To account for the highly inter-individual changes in menstrual cycle patterns, we used individually defined cycle phases as the backbone for further analysis in the NC group. This increases specificity as well as complexity, as the variation in cycle length between individuals leads to different lengths of the cycle phases. Linear mixed models were also used to assess cycle phase effects within participants on well-being domains and hormone levels, including a random intercept for every participant and menstrual cycle day as a random factor.

For the hormone levels, we calculated person-centered means and person-centered change scores. Change scores were calculated by subtracting each individual’s mean score from their daily scores. They are useful for understanding intra-individual variability, i.e., how each person’s psychological well-being and hormones fluctuate relative to their own baseline. These change scores help identify patterns and trends in an individual’s psychological well-being over time, independent of other individuals’ mood patterns.

To estimate the effect of hormones on each of the psychological well-being aspects we ran linear mixed models with cycle phase (5 levels), a random intercept for each individual, and NC day as a random factor. To examine whether the level of hormones or the change in hormones were related to the psychological well-being aspects, we conducted time-varying models separately for each hormone (estradiol, progesterone, and testosterone) using person-centered mean levels of hormones and person-centered change of hormones as fixed factors, resulting in a total of 9 psychological aspects * 3 types of hormones analyses. For each analysis, we also added interaction terms between cycle phase and both person-centered mean and person-centered change in hormone levels, to test whether the associations with hormones differed over cycle phases. In case of significant interactions, we stratified the dataset on cycle phase and reran the linear mixed model with person-centered hormone levels predicting psychological well-being variables.

In addition, network analyses were estimated in the NC group to examine the underlying associations between all psychological well-being aspects and hormonal levels (e.g. 12 nodes). Analyses were similar to the network models described above, but here, hormone levels were also added to the model. As an additional exploratory step, network analyses were estimated for each menstrual cycle phase, but these were not part of the main analyses due to small sample sizes and therefore included in the Supplementary Materials (Figure S2).

## Results

### Descriptives

As given in Table 1, the NC and OC groups had similar scores on many of the descriptive variables. However, more OC users were in a relationship more often and this group reported more sexual activity throughout the study period. NC participants more often reported using drugs during the study period compared to OC users. Whereas the majority of OC users reported their sexual orientation as straight/heterosexual (bisexual n = 1, queer n = 1), NC participants reported a wider variety of sexual orientations (bisexual n = 8, queer n = 4, asexual n = 2, pansexual n = 2, lesbian n = 1, and prefer to not disclose n = 1). All OC users described their gender identity as female/woman. For NC participants, 1 person identified as non-binary and another as gender fluid. Due to the synchronization of the study start date for NC participants, regardless of their menstrual cycle phase, the average salivary hormone levels on day 1 were similar between the OC and NC groups, likely reflecting the blurring of hormonal variations in the NC group. Supplementary Table S1 lists the OC formulations in our OC sample and shows all participants were currently using a combination OC and 67% of them used androgenic OCs.

**Table 1.** Sample characteristics

Characteristics	NC *N = 22*	OC *N = 18*	Statistic
Age, mean (SD)	19.7 (1.2)	20.2 (0.9)	F (1|38) = 1.42, p = .24
BMI, mean (SD)	21.9 (2.5)	20.9 (1.8)	F (1|38) = 2.02, p = .16
Cycle length, mean (SD), range	28.3 (2.5), 24–36	n/a	
Age of menarche, mean (SD)	12.8 (1.5)	12.4 (1.3)	F (1|38) = 0.97, p = .33
HADS – Anxiety, mean (SD)	11.1 (3.9)	9.6 (4.2)	F (1|38) = 1.31, p = .26
HADS – Depression, mean (SD)	6.0 (3.9)	5.2 (3.9)	F (1|38) = 0.45, p = .51
Relationship status			χ2(2) = 11.4, p < .001
Single	70%	40%	
In relationship	30%	60%	
Events during experiment			
Illness	0	0	n/a
Alcohol use	29%	28%	χ2(2) = 0.6, p = .43
Aerobic exercise	17%	20%	χ2(2) = 0.99, p = .18
Stressor	15%	15%	χ2(2) = 0.1, p = .49
Sexual activity	26%	49%	χ2(2) = 5.7, p = .01
Drug use	17%	10%	χ2(2) = 9.9, p = .001
Participation rate	96%	95%	χ2(2) = 0.5, p = .76
Hormone levels on day 1 of study			
Progesterone ng/mL, mean (SD)	21.2 (22.7)	9.1 (3.5)	F (1|34) = 3.3, p = .08
Estradiol ng/mL, mean (SD)	4.3 (1.9)	4.1 (1.3)	F(1|37) = 0.9, p = .77
Testosterone ng/mL, mean (SD)	17.7 (16.5)	11.6 (7.7)	F(1|37) = 1.8, p = .19

### Comparison between the OC and NC groups

**Figure 1.**
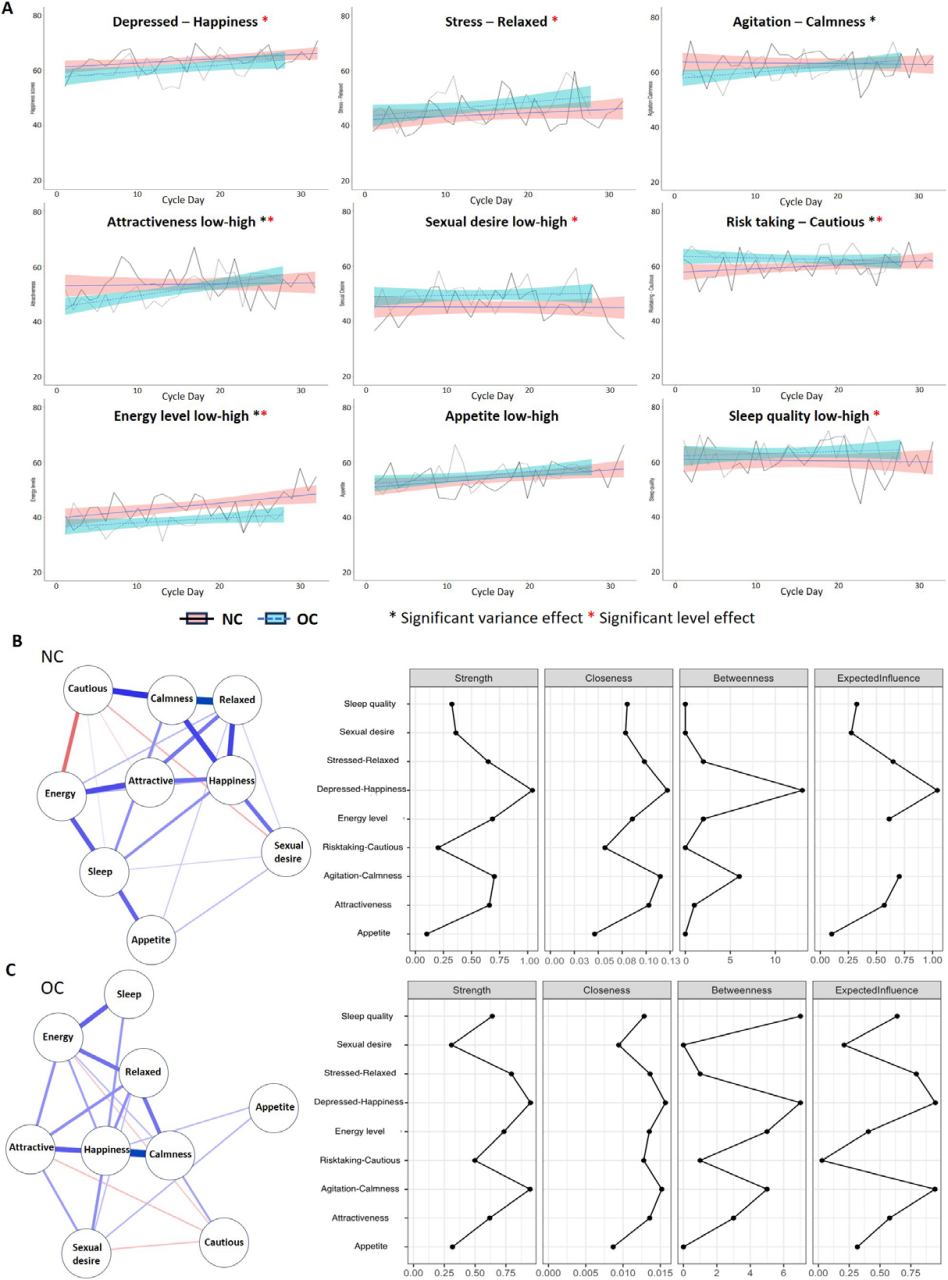
NC versus OC comparisons on psychological well-being levels, variation, and networks over 28 days. (A) Means and variation per well-being domain for NC and OC groups. OC users had significantly lower day-to-day variability in reports of agitation, attractiveness, risk taking, and energy levels. Data are shown for all menstrual cycle days and extend beyond 28 days for participants with longer cycles. (B) Network model of well-being domains for the NC group. (C) Network model of well-being domains for the OC groups. Network indices show strength, closeness, betweenness, and expected influence for each well-being domain. Blue lines represent a positive correlation and Red lines represent a negative correlation.

Figure 1A shows the mean and variation for the NC and OC groups separately for each psychological well-being domain. For the comparison of levels, NC participants showed significantly higher happiness/lower depression reports ((F(1|38,) = 4.27, p = .04, R^2^_m_ = .007), higher attractiveness ratings (F(1|38) = 4.24, p = .04, R^2^_m_ = .018), and energy levels (F(1|38) = 13.34, p < .001, R^2^_m_ = .010). OC users had lower reports of risk taking/more cautiousness (F(1|38) = 4.71, p = .03, R^2^_m_ = .009), more sexual desire (F(1|38) = 10.51, p < .001, R^2^_m_ = .010), and better sleep quality (F(1|38) = 3.93, p = .05, R^2^_m_ = .001). Lower reports of stress/more relaxed in OC users showed a non-significant trend (F(1|38) = 3.53, p = .06, R^2^_m_ = .001). No group differences were found for the domains of agitation/calmness (F(1|38) = 2.53, p = .11, R^2^_m_ = .009) or appetite (F(1|38) = 1.37, p = .24, R^2^_m_ = .001). Controlling for covariates, including age, BMI, alcohol use, and drug use, resulted only in insignificant group effects for depressed/happiness, sleep quality, and sexual desire (see Supplementary Table S2).

The Levene’s tests for variation showed significantly higher variation in NC participants compared to the OC participants for the reports on well-being domains of agitation/calmness (F(1|38) = 10.14, p < .001), risk taking/cautiousness(F(1|38) = 8.66, p = .003), attractiveness (F(1|38) = 8.71, p < .001), and energy levels (F(1|38) = 16.76, p < .001). A non-significant trend was found for lowered variability in the OC group for sleep quality (F(1|38) = 3.91, p = .05). No differences in variation were found for happiness/depressed (F(1|38) = 1.76, p = .18), stress/relaxed (F(1|38) = 1.05, p = .31), sexual desire (F(1|38) = 0.30, p = .58), and appetite (F(1|38) = 1.11, p = 0.29).

**Figure 2.**
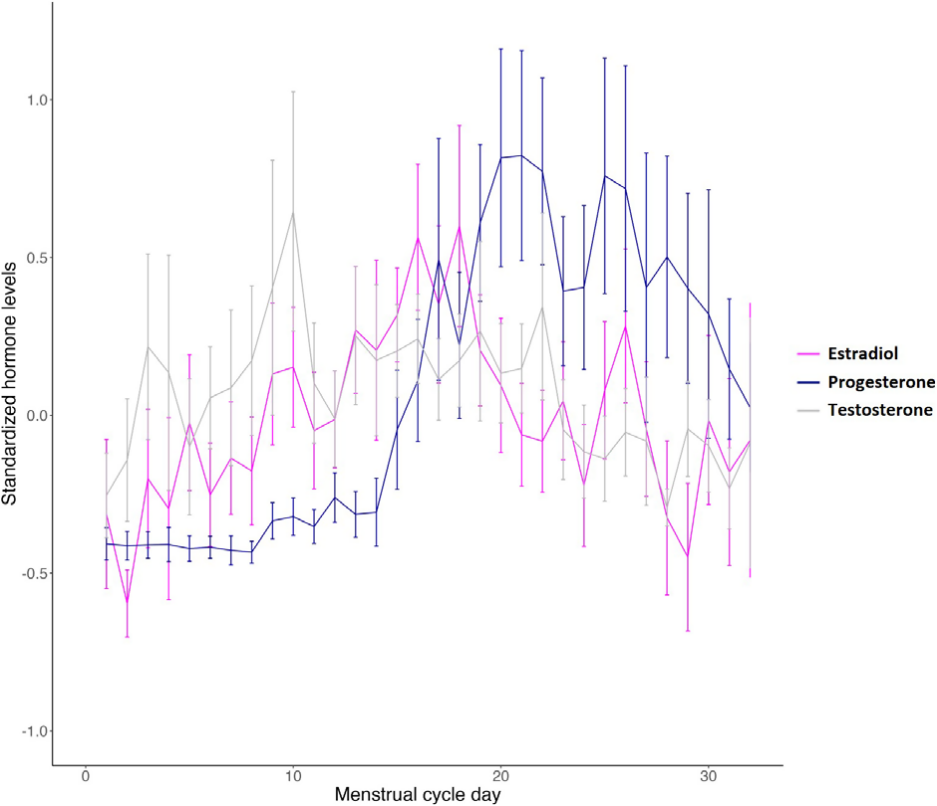
Standardized hormone levels across all menstrual cycle days, data extends beyond cycle day 28 for participants with longer cycles.

In Figures 1B and 2C, the network models are shown for the NC and OC groups. In the NC network, the number of non-zero edges in the sample was lower (22/36) than in the OC network (24/36), with a marginally larger mean weight of the sample (0.06) compared to the OC network (0.07). Therefore, the OC network is denser with a higher level of interaction between nodes and with stronger interactions than the NC network. Compared to the NC network, there was a larger role for sleep and cautiousness/risk taking in the OC network; however, happiness was the most central node in both networks.

### Menstrual cycle phase effects

We used the menstrual cycle phases (perimenstruation, mid-follicular, periovulation, early luteal, and late luteal) as the independent factor for a linear mixed analysis separately for all psychological and hormone aspects.

#### Hormone levels

Table 2 shows the average salivary hormone concentrations per cycle phase. Figure 2 depicts the standardized hormone levels averaged across participants for each menstrual cycle day. Note that this figure does not account for different cycle lengths for each of the participants. As can be seen, cycle phase had a significant effect on all hormones. Estradiol was highest during periovulation, progesterone was highest in the early luteal and mid-luteal phases, with testosterone highest in the periovulatory and early luteal phases.

**Table 2.** Definition and averages per cycle phase

	Peri menstruation	Mid-follicular	Periovulation	Early luteal	Mid-late luteal
Duration in days, mean (SD)	3.6 (1.2)	10.1 (2.7)	4.2 (1.4)	5.7 (3.1)	11.1 (5.3)
Estradiol^1^ *F(4|578*) = *7.83, p < .001*	4.1 (1.7)	4.6 (1.8)	5.6 (2.6)	5.0 (1.6)	4.8 (1.7)
Progesterone^2^ *F(4|572) = 34.07, p < .001*	12.3 (5.8)	11.6 (5.3)	14.2 (7.6)	30.2 (28.1)	35.0 (30.5)
Testosterone^3^ *F(4|577) = 6.72, p = .001*	15.3 (11.2)	16.1 (10.1)	21.0 (14.4)	18.4 (10.8)	15.9 (7.5)
Depressed – Happiness *F(4|599) = 1.85, p = .36, R^2^_m_ = .007*	61.1 (20.9)	63.7 (19.6)	60.4 (22.3)	65.2 (19.1)	63.3 (20.9)
Energy level low-high *F(4|599) = 0.83, p = .51, R^2^_m_ = .008*	41.4 (22.7)	45.1 (24.8)	40.5 (25.1)	44.8 (23.8)	45.0 (25.3)
Agitation – Calmness *F(4|599) = 1.24, p = .29, R^2^_m_ = .009*	62.9 (21.7)	63.1 (23.7)	60.1 (23.7)	66.5 (23.5)	61.0 (23.5)
Appetite low – high *F(4|599) = 2.75, p = .03, R^2^_m_ = .015*	55.7 (18.6)	52.6 (21.8)	48.7 (23.6)	51.6 (20.4)	57.3 (22.1)
Attractiveness low-high *F(4|599) = 3.27, p = .01, R^2^_m_ = .019*	47.6 (22.6)	56.1 (22.6)	52.3 (22.7)	57.5 (21.6)	52.7 (21.8)
Stress – Relaxed *F(4|599) = 1.65, p = .16, R^2^_m_ = .01*	45.5 (24.2)	41.9 (24.6)	44.0 (25.5)	49.1 (25.1)	43.2 (25.8)
Sexual desire low-high *F(4|599) = 1.10. p = .36, R^2^_m_ = .005*	39.6 (24.8)	45.0 (25.4)	47.3 (25.6)	45.1 (26.7)	45.3 (27.3)
Sleep quality low-high *F(4|599) = 1.38, p = .24, R^2^_m_ = .008*	56.9 (26.0)	63.2 (26.1)	59.8 (27.6)	61.6 (26.7)	57.6 (26.9)
Risk-taking – Cautious *F(4|599) = 0.46, p = .76, R^2^_m_ = .004*	57.0 (21.6)	58.9 (22.4)	60.0 (24.7)	61.5 (20.8)	60.3 (20.7)

Note. Shading indicates a significant main effect of Cycle Phase.

1 Significant higher levels of estradiol in periovulation and early luteal compared to perimenstrual and mid-follicular phases, all p < .05.

2 Significant higher levels of progesterone in early and mid to late luteal phases compared to perimenstrual, mid-follicular and periovulation phases, all p < .05.

3 Significant higher levels of testosterone in periovulation and early luteal phases compared to mid-late luteal and perimenstrual phases, all p < .05.

#### Psychological well-being throughout the cycle

In the mixed models without hormone levels, cycle phase significantly predicted the psychological well-being domains of appetite and attractiveness (Table 2). Post-hoc testing revealed participants reported the highest appetite in the mid-late luteal phase compared to the mid-follicular (mean difference = 4.59, 95% CI [0.06, 9.11]; p = .047), periovulatory (mean difference = 8.73, 95% CI [2.89, 15.57]; p = .003), and early luteal phase (mean difference = 5.79, 95% CI [0.64 to 10.94]; p = .03). Participants self-rated their attractiveness lowest in the perimenstrual phase compared to the mid-follicular (mean difference = −8.16, 95% CI [−13.97, −2.37]; p = .002) and early luteal (mean difference = −9.86, 95% CI [−16.20, −3.52]; p = .011) phases.

#### Psychological well-being and hormone levels throughout the cycle

To investigate whether the levels of hormones or the change of hormone levels within a cycle phase were related to psychological well-being, we ran time-varying linear mixed models. Both person-centered levels and within-person change levels were added to the mixed models separately for estradiol, testosterone, and progesterone. Supplementary Table S3 contains all main and interaction effects for each hormone and psychological well-being domain. Adding hormone levels to the analyses showed that cycle phase largely acted as a moderator for hormone effects on psychological well-being and indicated that the influence of hormones depends on the cycle phase they are measured in. This is further illustrated in Table 3, which shows the significant stratified analyses per cycle phase for hormone effects that significantly interacted with cycle phase. The interactive nature of cycle phase and hormone level is exemplified by the change in direction (positive/negative) of the hormone estimates.

**Table 3.** Stratified analysis for the interactions between cycle phase and hormone levels

	Perimenstruation	Mid-follicular	Periovulation	Early luteal	Mid-late luteal
**Testosterone**					
Depressed – Happiness *BP*	0.02 [−0.29, 0.52]	−0.01 [−0.32, 0.30]	−0.49 [−1.01,–0.01]	−0.10 [−0.55,0.36]	0.19 [−0.18, 0.57]
Risk taking – Cautious *BP*	−0.44 [−0.95, 0.06]	0.42 [0.06, 0.77]	−0.42 [−0.97, 0.12]	−0.29 [−0.73, 0.16]	0.10 [−0.29, 0.49]
**Estradiol**					
Stressed – Relaxed *BP*	−1.52 [−5.79, 2.76]	4.19 [1.28, 7.11]	−2.17 [−7.25, 2.92]	2.67 [−1.69, 7.04]	1.71 [−2.14, 5.55]
Sexual Desire low-high *BP*	5.63 [1.41, 9.84]	5.76 [2.79, 8.72]	2.88 [−2.22, 7.98]	0.13 [−4.55, 4.81]	−0.18 [−4.25, 3.89]
**Progesterone**					
Depressed – Happiness *BP*	−0.77 [−1.32, −0.22]	0.09 [−0.25, 0.43]	−0.27 [−0.92, 0.38]	−0.35 [−0.82, 0.12]	−0.79 [−1.19, −0.39]
Agitation-Calmness *BP*	−0.49 [−1.07, 0.10]	0.06 [−0.34, 0.46]	−0.76 [−1.42, −0.10]	−0.55 [−1.09,–0.01]	−1.09 [−1.55, −0.65]
Stressed – Relaxed *WP*	−0.20 [−0.66, 0.26]	−0.60 [−0.93, −0.26]	0.22 [−0.46, 0.91]	−0.05 [−0.26, 0.15]	−0.09 [−0.24, 0.07]
Energy low-high *BP*	0.29 [−0.33, 0.91]	−0.65 [−0.23, 1.08]	−0.14 [−0.86, 0.58]	−0.98 [−1.50, −0.45]	−0.57 [−1.08, −0.06]
Sleep quality low-high *WP*	0.24 [−0.26, 0.73]	−0.45 [−0.81, −0.09]	−0.46 [−1.19, 0.27]	0.14 [−0.07, 0.36]	−0.14 [−0.30, 0.03]
Attractiveness low-high *BP*	0.13 [−0.49, 0.75]	0.66 [0.28, 1.05]	0.61 [0.03, 1.24]	0.26 [−0 25, 0.76]	0.04 [−0.41, 0.48]
Sexual Desire low-high *BP*	0.51 [−0.16, 1.18]	0.74 [0.31, 1.18]	0.73 [0.02, 1.45]	0.30 [−0.32, 0.92]	−0.10 [−0.66, 0.46]
Risk-taking– Cautious B*P*	0.16 [−0.71, 1.02]	−0.52 [−1.12, 0.09]	−0.93 [−1.74, −0.13]	0.43 [−0.12, 0.97]	−0.43 [−0.88, −0.01]
Risk-taking– Cautious *WP*	0.12 [−0.48, 0.72]	−0.05 [−0.53, 0.43]	−1.00 [−0.18, −0.24]	−0.05 [−0.24, 0.14]	−0.02 [−0.15, 0.12]

*Note.* The values represent the estimate followed by the 95% confidence interval (CI) in square brackets. Shading indicates a significant main effect of Hormone, BP = between person-centered level effects, WP = within person-centered change effects.

It also shows that most psychological well-being domains were affected by Progesterone levels. More specifically, high levels and increases in progesterone levels were negatively associated with most well-being measures (Table 3). High progesterone levels were related to lower reports of happiness (in the perimenstrual and mid/late luteal phases), higher agitation/irritability (periovulatory, early, and mid/late luteal), more stress (mid-follicular), less energy (mid-follicular, early luteal, mid/late luteal), and decreased sleep quality (mid-follicular). Higher estradiol levels were associated with less stress (mid-follicular) and higher sexual desire (perimenstrual and mid-follicular). No significant effects of estradiol levels were found on periovulation. Higher testosterone levels were associated with lower risk taking in the follicular phase and lower happiness in the periovulatory phase.

**Figure 3.**
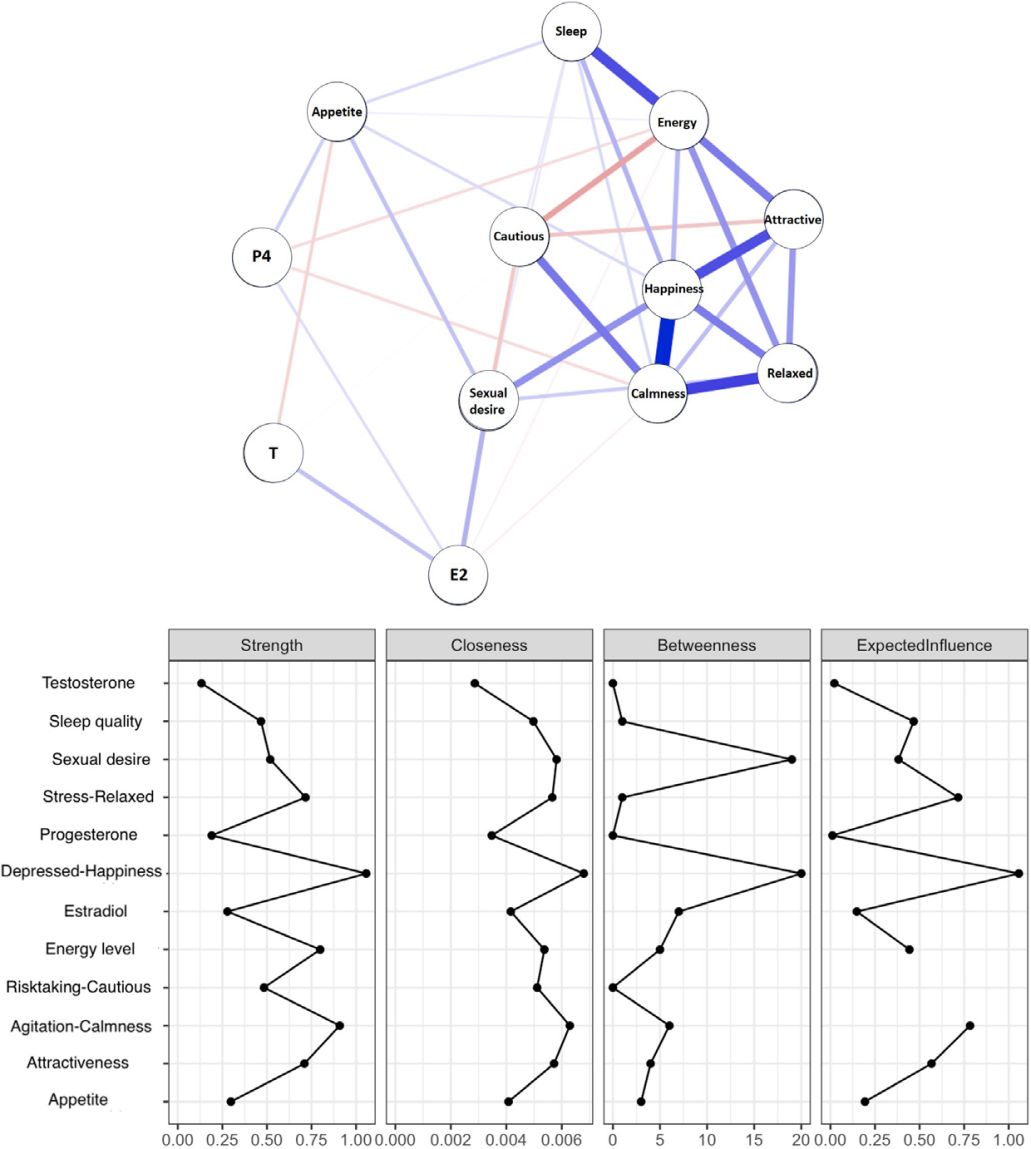
Network model and its characteristics of psychological well-being domains and hormone levels of estradiol (E2), progesterone (P4), and testosterone (T) in the NC group. Blue lines represent positive correlations and Red lines represent negative correlations.

Figure 3 shows the network model for the NC group to which hormone levels were added for the entire menstrual cycle (not considering cycle phase). For this model, there were 36/66 number of non-zero edges, with a mean weight of 0.04. The hormones estradiol, progesterone, and testosterone, together with self-reported appetite, had the lowest expected influence. The domains of relaxed-stressed, happiness-depressed, and calm-agitation had the highest expected influence. For exploratory purposes, network models were also created for each cycle phase separately, these are shown in the Supplementary Figure S2.

## Discussion

This study was designed to explore the dynamics of psychological well-being throughout the menstrual cycle, the contribution of sex hormones, and the possible alteration of this relationship in OC users. We found a lower variability in OC users compared to NC participants throughout 28 days for agitation/calmness, risk taking/cautiousness, attractiveness, and energy levels. This reduced variability was paralleled by lower overall ratings in OC users of happiness, attractiveness, risk taking, and energy levels, but they also showed higher reports of relaxation, sexual desire, and better sleep quality. The well-being network models indicated well-being domains to be more interrelated in the OC group than in the NC group, likely because the NC network was based on the entire 28-day period and menstrual cycle phase was not considered. In line with this, our results showed the relationship between hormone levels and psychological well-being in those with a natural cycle depended largely on menstrual cycle phase. For example, estradiol and progesterone were negatively related to sexual desire in the mid-late luteal phase but positively related in the other cycle phases. Likewise, progesterone was related to different well-being domains in the mid-late luteal phase compared to the mid-follicular phase. Analyzing hormone effects without considering specific cycle phases would therefore miss these nuanced interactions. These results fit with neuroimaging literature that shows sex hormone fluctuations throughout the cycle are related to structural and functional changes in brain areas responsible for affective and cognitive functioning (Dubol et al., 2021; Rehbein et al., 2021).

Lower day-to-day variation in affect has been brought forward as a possible mechanism underlying mental health side effects of OC users (Oinonen & Mazmanian, 2002). Our results confirm blunting, or at least lowered variability, on multiple psychological well-being domains, which was paralleled by negative overall effects of OCs on attractiveness, risk-taking, and energy levels. Importantly, OC users also reported a higher depressed mood. OC’s blunting has been reported regarding positive and negative affect (Hamstra et al., 2017; Oinonen & Mazmanian, 2002; Ott et al., 2008) but has not been studied outside of those domains. Whether reduced variability in psychological well-being has a stabilizing or detrimental effect likely depends on a person’s affect variability of dysregulation pre-OC use. For example, OC use may be beneficial for symptoms of PMS and PMDD (Freeman et al., 2012) and bipolar disorder (Rasgon et al., 2003) but may have adverse effects in those with a history of depression (Hall et al., 2012; Joffe et al., 2003). In our relatively healthy sample, our results do not show OC use to be associated with overall worsened psychological well-being; OC users had less stress, rated a higher sexual desire, and reported better sleep quality.

Whereas we found OC users to experience less stress, OC use is normally associated with an increased stress sensitivity as evidenced by a robust reduced psychosocial stress response (Gervasio et al., 2022). This puzzling effect is unlikely to be explained by external stressors, as we found no differences in the number of stressors experienced during the study between the groups. For sleep quality, studies have found both adverse (Bezerra et al., 2020; Morssinkhof et al., 2021) and beneficial (Guida et al., 2020) effects on sleep. In our study, OC use was associated with higher overall sexual desire compared to their NC counterparts. This contrasts with the reports of reduced sexual desire as one of the main OC side effects, which has been attributed to lowered free testosterone levels (Warnock et al., 2006). Our findings nevertheless match the majority of studies finding no differences or higher sexual desire after OC use, regardless of testosterone decreases (Warnock et al., 2006). The OC users were more often in a relationship, and even though analyses accounted for this factor, residual confounding cannot be excluded and may be the result of selection bias, where those who are in a relationship more often are inclined to use OCs.

Even so, these results highlight that hormone levels are not the sole determining factors of female psychological well-being, as external factors also have a powerful influence on resilience and risk. The positive effects of OC on overall well-being markers should also be considered in light of a possible survivor bias in our sample; those with adverse mood effects likely already discontinued their OCs and were not included in this study (Oinonen & Mazmanian, 2001). Regardless, our results suggest that when OCs are tolerated well enough to continue their use, their effects on well-being are small and can differ depending on the specific well-being domain. On the other hand, these effects on well-being levels and variability may increase the risk of affective and mood problems for those who are sensitive to the hormonal changes caused by OC use and should be studied further.

The increased variability in the NC group was also reflected in the network models, in which the OC group had higher strength and interconnectedness between well-being domains. This suggests that the natural hormonal fluctuations throughout the menstrual cycle might cause more variability and less predictability in how different well-being domains interact, while the stabilizing effect of OC use on hormonal levels may lead to a more tightly integrated experience of well-being. We found happiness to be the most influential factor when modeling hormones and psychological well-being in the menstrual cycle. Hormone levels were the least influential factor. Most likely, in the network models, the hormone levels only played a marginal role because these were modeled over the full menstrual cycle. Unfortunately, we did not have sufficient statistical power to estimate network models per cycle phase, but as can be seen in the Supplementary Materials (Figure S2), the influence of hormones seems to differ depending on cycle phases. In addition, we found that sexual desire, appetite, sleep, and energy are well-being domains that form a link between hormones and the more mood-related aspects. Hormonal effects on mood may, therefore, be mediated by these more physical markers of psychological well-being and should receive more attention when studying hormone-related mood changes.

In line with our results from the network models, we found most psychological well-being domains to depend on both cycle phase and hormone levels, mostly on progesterone levels*.* Not only did the hormones predict outcomes in different well-being domains depending on the cycle phase, but also the direction of the relationship between the same well-being domain and hormone also differed throughout the cycle. These results indicate that hormonal effects should be studied in the context of the specific menstrual cycle phases and cannot be generalized throughout the menstrual cycle, let alone to individuals with other hormonal statuses. On the other hand, we found more between-person level effects than within-person change effects of hormones on psychological well-being, regardless of the higher-powered within-person effects. This would argue for the usefulness of between-subject analyses while emphasizing rigorous cycle phase allocation.

Overall, higher levels of progesterone had expected negative effects, as we found that high progesterone was related to lower happiness, higher agitation/irritability, more stress, less energy, and decreased sleep quality reports. Consistent with these symptoms, the cyclical increase and administration of progesterone have been linked to increased amygdala reactivity to negative emotion induction (van Wingen et al., 2010; Bayer et al., 2013). Irritability is a core mood symptom of PMDD and one of the most commonly reported mood symptoms in the general population (Pearlstein et al., 2005; Studer et al., 2023). Likewise, tiredness has been listed among the top three symptoms reported during menstruation (Bruinvels et al., 2021; Schoep et al., 2019) and is a common physical symptom of PMS (Dennerstein et al., 2010). Positive effects of high levels of progesterone on attractiveness and sexual desire in the follicular and periovulatory phases, and on risk taking (periovulatory, mid/late luteal) were unexpected (Grebe et al., 2016; Jones et al., 2018; Roney & Simmons, 2013; Ziomkiewicz et al., 2012), but no prior study has ever looked at daily measurements per menstrual cycle phase. The beneficial effects of estradiol on stress and sexual desire confirmed results from previous studies (Jones et al., 2018; Ocampo Rebollar et al., 2017; Roney & Simmons, 2013) and correspond to estradiol’s stimulating effect on serotonin, oxytocin, and dopamine signaling (Bos et al., 2012; Frokjaer et al., 2015; Yoest et al., 2015). For testosterone, unexpected results showed higher levels to be associated with lower risk-taking in the follicular phase (Kurath & Mata, 2018) and lower happiness in the periovulatory phase (Giltay et al., 2012). These findings seem counterintuitive given testosterone’s established role in stimulating the reward response (Hermans et al., 2010). On the other hand, testosterone has been mostly studied using baseline levels rather than cycle-related changes. Perhaps higher baseline testosterone levels result in the absence of a periovulatory peak, or the expected peak was muddled by our method of cycle phase allocation, where the peak may have been divided into both the late periovulatory and early luteal phases. Overall, all these comparisons inflated the risk for Type I errors and should caution against any post-hoc inferences on the specific direction of cycle phase and hormone effects beyond the conclusion that the relationship between hormones and well-being depends on menstrual cycle phase.

Based on averages, our findings confirmed the lowest mean levels in different domains of well-being in the perimenstrual phase. Interestingly, our results hinted that so-called high-arousal negative states were more apparent in the mid/late luteal phase (high agitation, energy, and appetite) and low arousal symptoms in the perimenstrual phase (high depressed mood, and low energy, sexual desire). This would fit recent literature that posits hormone-related well-being to show different subtypes based on temporal, symptom, and underlying mechanistic characteristics. For example, a dimensional framework has been proposed based on PMDD or PME studies (Peters et al., 2024). In this framework, the mid-luteal phase with the estradiol and progesterone surge is characterized by symptoms of irritability and hyperarousal, whereas the perimenstrual hormone withdrawal is characterized by low mood and cognitive dysfunction. In contrast, the preovulatory estradiol surge is regarded as a period of less emotional vulnerability and increased reward sensitivity in the general population, supported by results from Blake et al. (2017) and Schiller et al. (2016).

### Strengths, limitations, and future directions

This relatively small sample has given insight into how hormonal markers that fluctuate throughout the cycle are sensitive to hormonal changes and how these differ in OC users. A major strength of this study is its inclusion of a wide range of psychological well-being variables sensitive to sex hormones, beyond negative and positive affect. Importantly, previous studies have found irritability and mood swings to be the main and most differentiating affective symptoms involved in PMS and PMDD (Pearlstein et al., 2005).

This study focused on a period of 28 days for daily measurements, based on the average length of a menstrual cycle combined with financial and practical feasibility. To understand intra-individual factors that may affect sensitivity to menstrual cycle changes, it would be necessary to include two or more cycles, which is already standard in PMDD and PME research (O’Brien et al., 2011). Using a 28-day cutoff means we were unable to capture a full menstrual cycle for people with a cycle length longer than 28 days. Yet, by having all participants start and end on the same day, the results were not affected by the reactive effects that often occur in intensive data-collecting methods. Namely, repeated reflection on target variables may influence the intensity, frequency, or quality of the variable. In addition, synchronizing the experiment’s timeline for all participants likely contributed to the high retention rates.

Cycle phase allocation of the follicular phases can be done using the forward and backward counting method used in this study, but the golden standard is to use LH testing or measures of basal body temperature to determine the day of ovulation (Schmalenberger et al., 2021). This means we were unable to control for anovulatory cycles, and as the cycle following anovulation can still present with bleeding, it is easy to go unnoticed. However, we found only 2 participants without a clear progesterone peak in their daily salivary hormone levels, which would indicate a limited influence of anovulatory cycles and promote the validity of our hormone measures. In addition, taking saliva samples in the morning meant that the testosterone levels were assessed at their highest concentration due to their circadian rhythm (Al-DujailI & Sharp, 2012). Within-person variance was limited by instructing participants to take the samples at the same time every day. Even though sex hormones are relatively unstable and the collection and storage relied, at least partially, on the participants themselves, we found expected cycle phase changes in hormone levels. This indicates that salivary hormones provide an accessible (in terms of feasibility and costs) way to assess the much-needed hormonal contributions in dense sampling approaches.

We found our NC group to have more variation in gender identity and sexual orientation, which may differentially impact mental health (likely through (social) stressors) (Herek & Garnets, 2007; ‘The Health of Lesbian, Gay, Bisexual, and Transgender People: Building a Foundation for Better Understanding’, 2011). These demographics often go unreported in the current literature, but our results show the potential and importance of including questions on gender identity and sexual orientation. This would not only serve to account for selection bias, but also improve (clarity on) generalizability of the results. The impact of these differences between and within groups should be taken into account in future studies with larger samples. In addition, the OC group was too small to assess the effects of relevant characteristics regarding OC use on fluctuations in psychological well-being. For example, standardized use of OCs involves a pill-pause week where users stop taking the pill for 4–7 days. During this phase, synthetic hormone levels reduce, and endogenous hormone production increases which can result in a withdrawal bleed. Recent research indicates that this pill pause negatively influences affective processing and mood (Noachtar et al., 2023; Radke & Derntl, 2016). This and other OC-specific characteristics may also affect mood-related side effects, including OC androgenicity (Dhont, 2010) (Schaffir et al., 2016) and should be accounted for in future studies.

The small effect size of the menstrual cycle and OC use on psychological well-being should be understood in the context of our healthy and high-functioning sample at a university campus. In a follow-up study, we aim to assess the influence of the severity of self-reported PMS symptoms as a measure of hormone sensitivity, and whether it accounts for inter-individual differences in hormone-related well-being fluctuations. In addition, we will address the intra-individual differences and stressors that relate to clinically relevant makers of depression and anxiety over the cycle. Moreover, the relationship between hormones and psychological well-being fluctuations in individuals assigned either male or female at birth will be compared. With this approach, we aim to address the persistent stereotype that hormone-related well-being is more variable when you have a menstrual cycle. This argument is often used to argue for excluding female participants in research (Rechlin et al. 2022). However, more research is proving this argument to be unfounded (Levy et al., 2023; Weigard et al., 2021).

Finally, this study applied a network model to study the dynamic and time-varying nature of the field of female psychoneuroendocrinology. Network models provide a methodology to map the relationships between symptoms, how they differ between (groups of) people and over time, and they are particularly informative when studying transdiagnostic, dynamic mechanisms (Roefs et al., 2022). Network models have been applied to studies of mental disorders and offer a new and promising opportunity in the field of psychoneuroendocrinology. Our results have given a first exploration of how networks change throughout the cycle, but future studies should expand on menstrual cycle phase-specific networks to capture the varying dynamics of hormone-symptom associations in the menstrual cycle.

## Conclusion

Our results highlight the necessity and opportunities of considering hormonal status when studying psychological well-being and emphasize that associations between hormones and psychological well-being differ according to menstrual cycle phases. This study provides further support that emotional blunting may be the mechanism that can explain OC-related side effects, dependent on pre-OC use psychological functioning. An increased understanding of the nuance and complexity behind hormone-related mental health will add to the psychoeducation of women and those assigned female at birth and inform research. It will also aid in increasing menstrual (mental) health literacy and awareness, which can go hand in hand with increased general health and well-being (Cunningham et al., 2024).

## Supporting information

Doornweerd and Gerritsen supplementary material Doornweerd and Gerritsen supplementary material

## Supplementary material

The supplementary material for this article can be found at http://doi.org/10.1017/S003329172400357X.
