# Supplementary material for: 28 days later: A prospective daily study on psychological well-being across the menstrual cycle and the effects of hormones and oral contraceptives
Source: Psychol Med. 2025 Feb 7;55:e19. doi: 10.1017/S003329172400357X (PMC12017372; doi:10.1017/S003329172400357X)
Supplement: Doornweerd and Gerritsen supplementary material [file S003329172400357Xsup001.docx]

**Supplementary material**

Menstrual cycle phase allocation

The **mid-follicular** **phase** (low progesterone, slightly rising estradiol levels) was defined as days +3 to days -14. The **periovulatory phase** (strong rise and fall of estradiol and testosterone, a slight increase of progesterone) was phased as days -15 to -12. The **early luteal phase** (rising estradiol and progesterone levels) was defined as the days -13 until the day of the observed progesterone peak (based on salivary samples). The **mid/late luteal** **phase** (peaking and falling progesterone and estradiol levels), was matched to the days from the progesterone peak to day -3. The **perimenstrual** **phase** (low estradiol and progesterone levels) was defined as days -2 to the days that fell within the first 10% of the individual’s menstrual cycle.


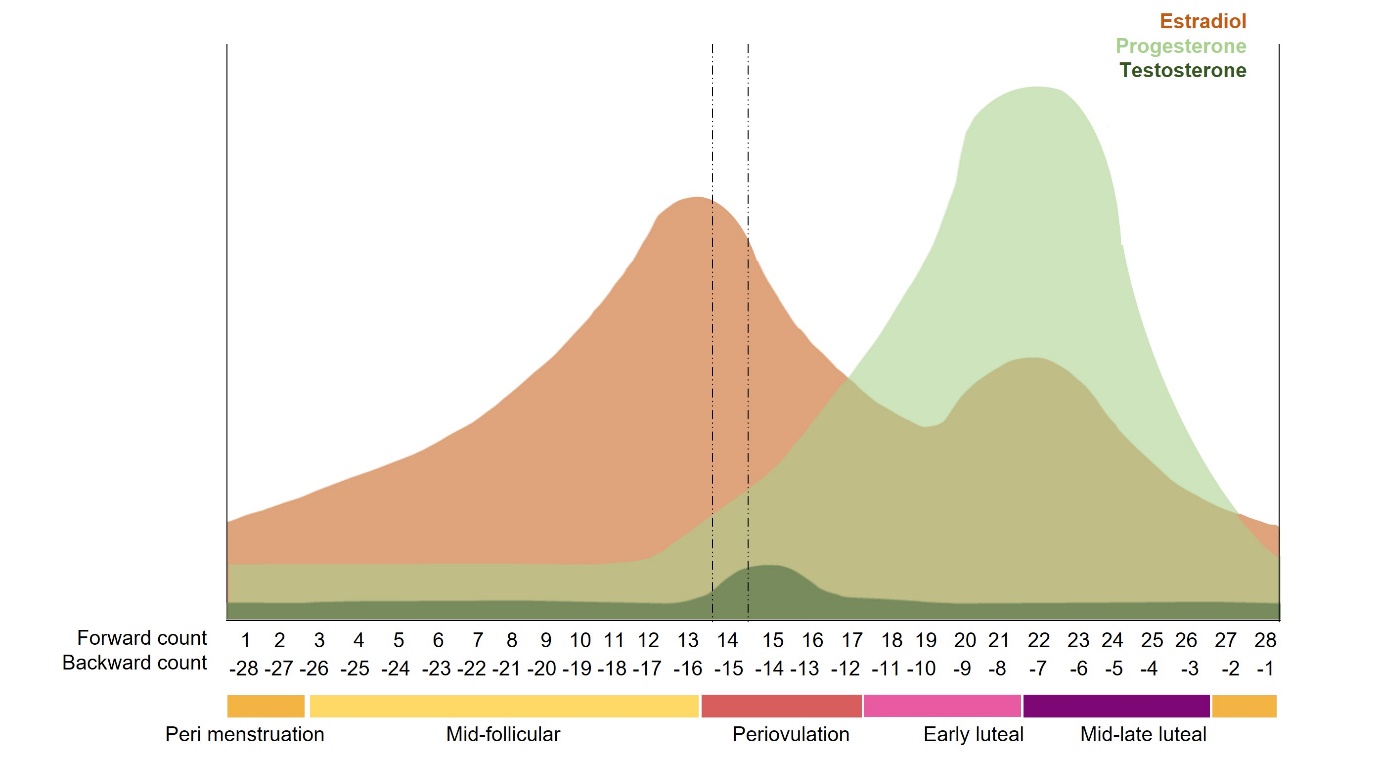


**Fig. S1**. Representation of the study design and menstrual cycle phases included in this study using a typical 28-day ovulatory menstrual cycle with fluctuating levels of estradiol, progesterone, and testosterone. Five menstrual phases (perimenstrual phase, mid-follicular, periovulatory, early luteal, and mid-late luteal) were selected as temporal windows of interest for analysis.

**Table S1.** OC formulations by androgenicity

| **OC formulations** | | **n** | | **Androgenicity** |  |
| --- | --- | --- | --- | --- | --- |
| Microgynon (0.03/0.15 mg ethinylestradiol/levonorgestrel) | | 9 | | Androgenic |  |
| Lovette (0.02/0.10mg ethinylestradiol/levonorgestrel) | | 3 | | Androgenic |  |
| Yasmin (3/0.03mg drospirenon/ethinylestradiol) | | 1 | | Anti-androgenic |  |
| Yaz (3/0.02mg drospirenon/ethinylestradiol) | | 2 | | Anti-androgenic |  |
| 0.03/2 mg ethinylestradiol/dienogest | | 1 | | Anti-androgenic |  |
| Qlaira - multiphasic | | 2 | | Anti-androgenic |  |
| 2 mg estradiolvalerate | |  | |  | |
| 2/2 mg estradiolvalerate/dienogest | |  | |  | |
| 2/3 mg estradiolvalerate/ dienogest | |  | |  | |
| 1 mg estradiolvalerate | |  | |  | |

**Table S2.** Full adjustment models for group comparison on well-being levels

|  | **NC vs OC*** | **Age** | **BMI** | **Alcohol use** | **Drug use** |
| --- | --- | --- | --- | --- | --- |
| Depressed – Happiness | 2.93 p = .15 | 0.06 p = .95 | 0.52 p = .26 | 0.39 p = .77 | 0.15 p = .93 |
| Stress – Relaxed | -3.37 p = .17 | 0.34 p = .75 | 1.82 p <.001 | -2.16 p = .18 | 8.82 p <.001 |
| Agitation – Calmness | 1.36 p = .54 | -3.83 p <.001 | -0.93 p = .06 | -0.894 p = .53 | 1.09 p = .58 |
| Sleep quality low-high | -2.07 p = .27 | 0.34 p = .76 | -0.27 p = .63 | -1.65 p = .36 | 1.3 p = .60 |
| Appetite low – high | -1.64 p = .43 | 0.18 p = .84 | 0.34 p = .76 | -1.66 p = .26 | 1.45 p = .48 |
| Sexual Desire low-high | -3.66 p = .11 | 3.46 p = .002 | 0.26 p = .64 | -1.19 p = .46 | 2.15 p = .33 |
| Risk-taking – Cautious | -5.99 P = .007 | -3.4 p <.001 | 0.14 p = .78 | -3.88 p = .007 | -5.26 p = .007 |
| Attractiveness low-high | 5.92 p = .007 | 0.36 p = .71 | 0.52 p = .28 | -0.20 p = .89 | -4.55 p = .019 |
| Energy level low-high | 4.96 p = .03 | 0.73 p = .47 | 0.45 p = .38 | -2.84 p = .07 | 0.60 p = .78 |

* positive estimate = NC> OC, negative estimate = NC< OC

**Table S3.** Stratified analyses for hormone levels

|  | **Cycle Phase** | **BP Hormone Level** | **Cycle Phase * BP** | **WP Hormone Change** | **Cycle Phase * WP** |
| --- | --- | --- | --- | --- | --- |
| **Estradiol** |  |  |  |  |  |
| Depressed - Happiness | 2.87 p = .58 | 1.04 p = .31 | 5.11 p = .28 | 0.06 p = .80 | 2.18 p = .70 |
| Agitation - Calmness | 2.54 p = .64 | 0.16 p = .68 | 1.56 p = .82 | 0.16 p = .68 | 4.63 p = .33 |
| Stress - Relaxed | 9.25 p = .06 | 0.31 p = .58 | 9.44 p = .05 | 0.22 p = .64 | 0.33 p = .99 |
| Energy level low - high | 5.42 p = .25 | 3.23 p = .07 | 7.16 p = .13 | 1.17 p = .28 | 1.40 p = .84 |
| Sleep quality low - high | 2.20 p = .70 | 0.40 p = .53 | 1.51 p = .82 | 0.08 p = .77 | 7.38 p = .12 |
| Appetite low - high | 3.54 p = .47 | 0.57 p = .44 | 3.25 p = .52 | 2.63 p = .10 | 5.21 p = .27 |
| Attractiveness low - high | 0.39 p = .98 | 0.10 p = .76 | 0.40 p = .98 | 0.22 p = .64 | 4.04 p = .40 |
| Sexual Desire low - high | 14.19 p = .007 | 2.46 p = .12 | 13.26 p = .01 | 0.15 p = .69 | 4.33 p = .36 |
| Risk taking - Cautious | 7.36 p = .12 | 0.06 p = .81 | 7.63 p = .11 | 0.55 p = .46 | 4.78 p = .31 |
| **Testosterone** |  |  |  |  |  |
| Depressed - Happiness | 4.47 p = .35 | 0.03 p = .86 | 1.1 p = .03 | 1.21 p = .27 | 5.73 p = .22 |
| Agitation - Calmness | 11.01 p = .03 | 0.26 p = .61 | 7.51 p = .11 | 0.00 p = .95 | 3.09 p = .54 |
| Stress - Relaxed | 3.63 p = .45 | 0.25 p = .62 | 2.27 p = .69 | 2.90 p = .08 | 4.94 p = .29 |
| Energy level low - high | 7.07 p = .13 | 1.46 p = .23 | 8.13 p = .09 | 0.06 p = .80 | 0.92 p = .92 |
| Sleep quality low - high | 1.54 p = .82 | 0.01 p = .91 | 2.05 p = .72 | 5.55 p = .02 | 6.36 p = .17 |
| Appetite low - high | 5.57 p = .23 | 0.89 p = .34 | 3.46 p = .48 | 0.37 p = .54 | 5.75 p = .22 |
| Attractiveness low - high | 13.94 p = .007 | 1.08 p = .30 | 7.33 p = .11 | 0.09 p = .77 | 3.96 p = .41 |
| Sexual Desire low - high | 3.90 p = .42 | 0.05 p = .82 | 3.94 p = .41 | 0.50 p = .48 | 1.30 p = .86 |
| Risk taking - Cautious | 12.50 p= .01 | 1.50 p = .22 | 12.64 p = .01 | 0.14 p = .71 | 2.86 p = .58 |
| **Progesterone** |  |  |  |  |  |
| Depressed - Happiness | 16.18 p= .003 | 1.06 p = .30 | 12.90 p = .01 | 1.77 p = .18 | 3.69 p = .44 |
| Agitation - Calmness | 1.26 p = .04 | 0.37 p = .54 | 11.26 p = .02 | 0.36 p = .56 | 2.02 p = .73 |
| Stress - Relaxed | 6.98 p = .14 | 0.48 p = .49 | 5.00 p = .28 | 0.02 p = .90 | 9.38 p = .05 |
| Energy level low - high | 15.11 p = .004 | 0.18 p = .66 | 11.67 p = .02 | 0.06 p = .81 | 2.16 p = .71 |
| Sleep quality low - high | 1.94 p = .75 | 0.32 p = .57 | 2.28 p = .68 | 2.20 p = .13 | 9.90 p = .04 |
| Appetite low - high | 6.24 p = .18 | 0.07 p = .79 | 5.30 p = .26 | 0.01 p = .91 | 4.86 p = .30 |
| Attractiveness low - high | 7.40 p = .12 | 0.02 p = .89 | 9.91 p = .04 | 0.05 p = .83 | 1.64 p = .80 |
| Sexual Desire low - high | 17.85 p = .001 | 0.11 p = .73 | 19.02 p < .001 | 0.09 p = .77 | 5.42 p = .25 |
| Risk taking - Cautious | 11.12 p = .03 | 0.57 p = .45 | 15.21 p = .004 | 1.14 p = .29 | 9.61 p = .05 |

*Note.* Values shown are F and p values for every test variable. Numerator df values: Cycle Phase = 4, BP Level = 1, WP Change = 1, Interactions = 4. Denominator is for all F-tests: 607. BP = between person-centered level effects, WP = within person-centered change effects.


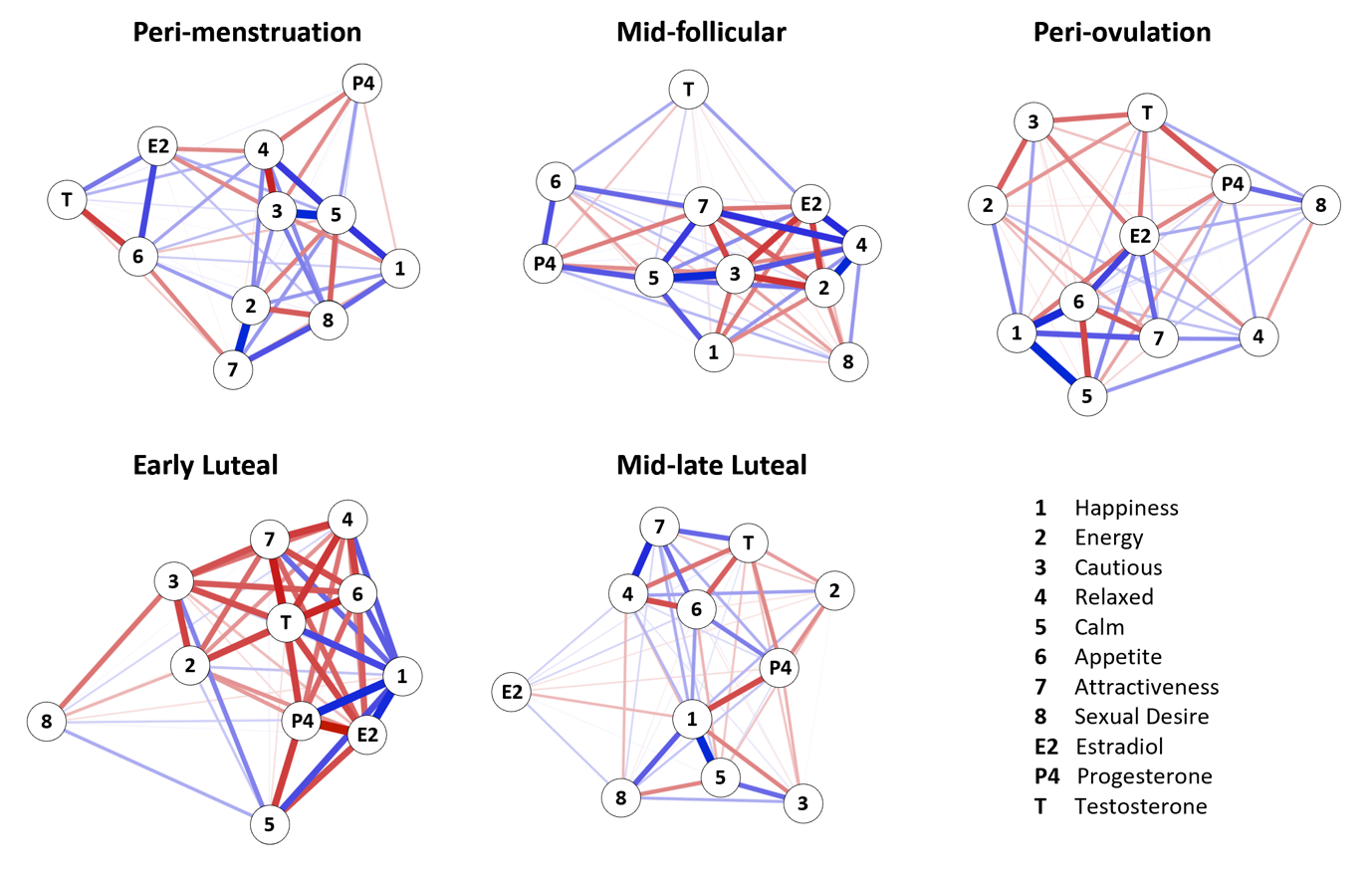


**Fig. S2**. Network models per cycle phase. Red = negative and blue is positive correlation
